# Time-series analysis of the association between air pollution exposure and outpatient visits for dry eye disease: a case study in Zhengzhou, China

**DOI:** 10.3389/fpubh.2024.1352057

**Published:** 2024-03-14

**Authors:** Mengting Xia, Yingrui Yang, Jiali Sun, Ranran Huang, Yonghui Huang, Mengqi Zhang, Xi Yao

**Affiliations:** ^1^Henan Eye Institute & Henan Eye Hospital, Henan Provincial People’s Hospital, People’s Hospital of Zhengzhou University, Zhengzhou, China; ^2^Hospital-Acquired Infection Control Department, School and Hospital of Stomatology, Wenzhou Medical University, Wenzhou, China

**Keywords:** dry eye disease, generalized additive model, air pollution, outpatient visits, ocular surface disease

## Abstract

**Background:**

Dry eye disease (DED) is a prevalent ocular surface disease that significantly impacts patients’ quality of life. The association between air pollution and the risk of dry eye disease remains uncertain.

**Methods:**

Data on outdoor air pollutants, meteorological information, and outpatient visits for DED were collected from July 1, 2014, to December 31, 2019. The relationship between ambient air pollutants and DED outpatient visits was analyzed using a generalized additive model with a Poisson distribution.

**Results:**

Among the 5,204 DED patients included in the study, 63.76% were female and 36.24% were male. The single-pollutant model revealed a significant association between a 10 μg/m^3^ increase in concentrations of fine-particulate matter with a median aerometric diameter of less than 10 μm (PM_10_), sulfur dioxide (SO_2_), nitrogen dioxide (NO_2_), and carbon monoxide (CO) and outpatient visits for DED. Fine-particulate matter with a median aerometric diameter of less than 2.5 μm (PM2.5) showed a significant association with DED outpatient visits in males and the 19–59 years age group. The strongest associations between air pollutants and outpatient visits were observed in male patients and during the cold season.

**Conclusion:**

The noteworthy correlation between air pollutants and DED outpatient visits can offer evidence for policy makers and underscore the significance of reinforcing environmental protection.

## 1 Introduction

Dry eye disease (DED) is a prevalent ocular surface disease that presents with symptoms such as ocular surface discomfort, fatigue, impaired vision, and tear film instability, significantly impacting the quality of life of million of individuals globally (1). DED is a multifactorial ocular surface disease caused by an imbalance in tear film homeostasis, resulting in tear film hyperosmolarity, instability, ocular surface inflammation and injury, as well as neurosensory abnormalities (2). Common symptoms of DED encompass excessive mucus production, tear film evaporation impairment, itching, burning, light sensitivity-induced redness, and eyelid movement difficulty (3). From 1997 to 2021, the estimated global prevalence of DED was 11.59%, with East Asia exhibiting the highest prevalence at 42.8% (4). Therefore, DED has emerged as a significant and escalating public health concern.

Air pollution is a significant global public health issue, as evidenced by numerous epidemiological studies demonstrating its association with a substantial increase in outpatient visits (5) and mortality rates (6). However, the majority of studies have primarily concentrated on the respiratory system’s response to air pollution, leaving the impact on ocular health relatively neglected. Extended exposure of ocular surfaces to air pollutants heightens their vulnerability to these substances (7). Both air pollutants and weather conditions have the potential to impact the health of the tear film and ocular surface, disrupting the eye’s natural lubrication and protective mechanisms. Prolonged exposure to air pollution can result in various symptoms of ocular surface diseases, such as ocular discomfort, abnormal tear film structure, and inflammation of the ocular surface (8–10). The extent to which long-term exposure to high levels of air pollution affects the eye is not fully understood. Nevertheless, heightened levels of air pollution might be linked to greater instability of the tear film (11, 12). The association between air pollution and allergic conjunctivitis has been confirmed (5, 13). Allergic conjunctivitis, characterized by the release of numerous inflammatory mediators, leads to deficiencies in the mucus layer and instability of the tear film, making it a major contributing factor to DED (14). Limited research has been conducted on the prolonged exposure of the ocular surface to outdoor air pollutants. Only a handful of studies have explored the impact of air pollution on the ocular surface (9, 15–19). Ocular surface abnormalities associated with air pollution are regarded as a subset of DED due to their distinctive features, including tear film abnormalities, elevated ocular surface disease index, and reduced conjunctival goblet cell density (9, 17, 18). The majority of studies investigating the impact of air pollution on the ocular surface have been limited by short study durations and a narrow range of air pollutants examined, potentially contributing to inconsistent findings influenced by factors such as geographical location and population disparities. This study examined the relationship between outdoor air pollutants, including fine-particulate matter with a median aerometric diameter of less than 2.5 μm (PM_2.5_), fine-particulate matter with a median aerometric diameter of less than 10 μm (PM_10_), sulfur dioxide (SO_2_), nitrogen dioxide (NO_2_), carbon monoxide (CO), ozone (O_3_) and outpatient visits for DED in Zhengzhou, China. The study also accounted for potential confounding meteorological factors. This study establishes a foundation for understanding the link between air pollution exposure and dry eye disease, thereby supporting the development of enhanced prevention and intervention strategies.

## 2 Materials and methods

### 2.1 Study area

**Figure 1:**
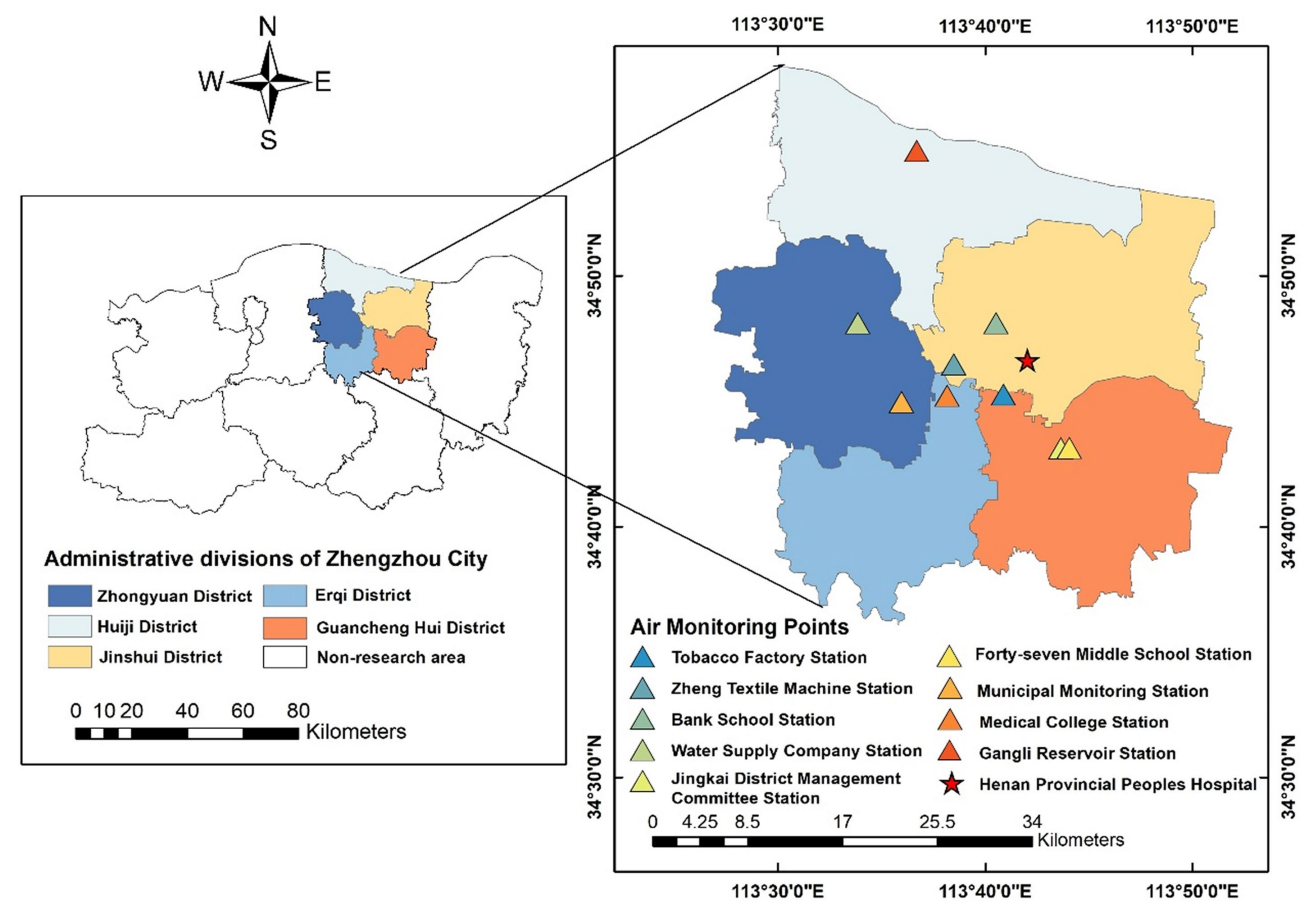
Locations of environmental monitoring stations and hospitals in Zhengzhou, China.

Zhengzhou is a significant central city and a comprehensive transportation hub in central China. Situated south of the North China Plain, north of central Henan Province, and downstream of the Yellow River, Zhengzhou serves as both the capital of Henan Province and the core city of the Central Plain Urban Agglomeration. Zhengzhou experiences a northern temperate continental monsoon climate, characterized by frequent shifts between cold and warm air masses, as well as four distinct seasons: spring, summer, autumn, and winter. Our study area is the five primary urban areas of Zhengzhou City (Figure 1). Zhengzhou is administratively divided into six districts: Zhongyuan, Erqi, Huiji, Guancheng Hui, Jinshui and Shangjie. Shangjie District, situated in the northwest and distant from the city center, was excluded from the study due to the inaccessibility of hospitals, pollution monitoring stations, and data accuracy concerns.

### 2.2 Data sources

The disease data were collected from the Henan Eye Hospital, which is a branch of the Dry Eye and Ocular Surface Diseases Center and serves as the National Level Dry Eye Demonstration and Guidance Center. The patient information regarding dry eye visits to this hospital was obtained from the hospital information management system for the period of July 1, 2014, to December 31, 2019. The majority of patients who visited the dry eye clinic at the ophthalmology department of the hospital were residents of Zhengzhou. Patients residing outside the study area were excluded to ensure that the study subjects were part of the permanent population within the study area. The diagnosis of DED was based on the standardized International Classification of Diseases-10th Revision (ICD-10) code for ophthalmology, specifically H11.103. We reviewed outpatient medical records and conducted ophthalmic examinations to confirm the diagnosis of DED and related complaints. The final diagnostic information was reviewed by either a mid- or senior-level physician in the department or the attending physician with expertise in the field to ensure the accuracy of diagnosis and data classification. The dataset only contains basic outpatient information, patient identification numbers (each patient has a unique number for identification purposes), visit dates, age, gender, and home addresses (including ZIP codes). Informed consent was not required because the data came from anonymous datasets typically used for administrative purpose.

Meteorological data including average daily temperature, average daily pressure, average daily wind speed, and average daily relative humidity in Zhengzhou City from July 1, 2014 to December 31, 2019 were obtained from the National Meteorological Science Data Center.^1^

Air pollution data were collected from nine national air monitoring stations: Tobacco Factory Station, Zheng Textile Machine Station, Bank School Station, Water Supply Company Station, Jingkai District Management Committee Station, Forty-seventh Middle School Station, Municipal Monitoring Station, Medical College Station, and Gangli Reservoir Station (Figure 1). The daily average concentrations of PM_2.5_ and PM_10_ were measured using the tapered element oscillating microbalance (TEOM) method. Ultraviolet fluorescence and chemiluminescence methods were used to measure SO_2_ and NO_2_. An infrared (IR) analyzer and a non-dispersive ultraviolet fluorescence photometer were employed to measure CO and O_3_, respectively. Air pollutant concentrations at each monitoring site were collected from July 1, 2014, to December 31, 2019. The pollutants included PM_2.5_, PM_10_, SO_2_, NO_2_, CO, and O_3_. Hourly concentrations were recorded for PM_10_, SO_2_, NO_2_, and CO, with daily averages calculated. O_3_ concentration was determined as the 8-h maximum. The air pollution data are stored in the database along with daily meteorological data and outpatient visit data.

### 2.3 Data analysis

We used the Spearman correlation coefficient to assess the correlation between air pollutants and meteorological factors. Daily outpatient visits for DED are rare events that approximately follow a quasi-Poisson distribution. To assess the effect of air pollutant exposure on DED outpatient visits, we employed a combination of a quasi-Poisson generalized additive model (GAM) and a distributional lag nonlinear model (DLNM) (20). All models control for the effects of confounding variables, including meteorological factors, by smoothing using natural cubic spline curves (ns) with three degrees of freedom (df). Dummy variables were used to control for variables that may have potential effects, such as day-of-week and holiday effects. To assess the potential lagged effects of air pollutants, we defined same-day exposure as lag 0 and considered a maximum lag period of 7 days. We separately examined the effects of single-day lags (lag 0 to lag 7) and cumulative lags (lag 0–1 to lag 0–7) of air pollution. The choice of df is determined based on the principle of residual independence, which selects the minimum value of the sum of the absolute values of the partial autocorrelation function (PACF) of the residuals from the base model. The final model is shown below:


$$Y_{t}\sim \mathrm{quasiPoisson}\left(\mu_{t}\right)$$

$$\log\left(\mu_{t}\right)=\beta X_{t}+\mathrm{ns}\left(t,df_{t}\right)+\mathrm{ns}\left(Z_{t},df_{t}\right)+DOW+\alpha$$

The variables in the model are defined as follows: $Y_{t}$ i represents the actual number of hospital admissions on day *t*; $\mu_{t}$ represents the expected value of hospital admissions on day *t*; $X_{t}$ represents the concentration of air pollutants (PM_2.5_, PM_10_, CO, SO_2_, NO_2_, O_3_) on day *t*; β is the regression coefficient; ns represents the natural cubic spline; $df_{t}$ represents the degrees of freedom; $Z_{t}$ represents the meteorological factors (mean temperature, mean air pressure, mean relative humidity, and mean wind speed) on day *t*; DOW represents the weekly dummy variable, and α is a constant term. The lag model determines the optimal lag period based on the maximum odds ratio and the minimum *p*-value. Sensitivity analyses of the main results were conducted by varying the *df* of the time variable and the degrees of freedom of the mean temperature, air pressure, relative humidity, and wind speed. Statistical significance was defined as a *p*-value less than 0.05.

To investigate potential modifying factors, patients were categorized into two gender groups (male and female) and three age groups (0–18, 19–59, and ≥ 60 years). Additionally, considering the seasonal characteristics of Zhengzhou, the year was divided into two seasons: the warm season (April to September) and the cold season (January to March and October to December).

## 3 Results

Table 1 presents the descriptive statistics of daily outpatient visits for DED at Henan Provincial Eye Hospital, including meteorological factors. Among the 5,204 outpatient visits for dry eye syndrome, there were 1886 visits (36.24%) by males and 3,318 visits (63.76%) by females. Regarding age groups, there were 315 visits (6.05%) in the 0–18 age group, 2,546 visits (48.92%) in the 19–59 age group, and 2,343 visits (45.02%) in the 60 and above age group. Among the 5,204 DED patients included in this study, females accounted for a larger proportion (63.76%) compared to males. Patients aged 0–18 years constituted the smallest age group (6.05%), and the warm season had a slightly higher seasonal distribution (52.67%) compared to the cold season. The average values of mean temperature, air pressure, relative humidity, and wind speed were 16.44°C, 1045.0 hPa, 62.21%, and 2.52 m/s, respectively.

**Table 1:** Descriptive statistics for meteorological variables, and dry eye disease visits from July 1, 2014, to December 31, 2019, in Zhengzhou, China.

Variables	Number of measurements	Min	P25	P50	P75	Max	Mean	SD
Meteorological factors								
Mean temperature (°C)	2010	0	8	17	25	34	16.44	9.38
Air pressure (hPa)	2010	128	1,008	1,017	1,025	1,011	1,045	42.07
Relative humidity (%)	2010	16	50	63	76	97	62.21	17.28
Wind speed (m/s)	2010	0	2	2	3	9	2.52	1.06
No. of visits for DED								
Total	5,204	0	1	2	4	25	3	3
Gender								
Male	1886	0	0	0	2	10	0.94	1.35
Female	3,318	0	0	1	1	15	1.65	1.99
Age (years)								
0–18	315	0	0	0	0	6	0.16	0.46
19–59	2,546	0	0	1	2	11	1.27	1.61
60-	2,343	0	0	1	2	20	1.17	1.90
Season								
Warm (April to September)	2,741	0	1	2	4	17	2.72	2.76
Cold (October to March)	2,463	0	0	1	3	25	2.46	3.02

P25, P50, and P75 denote the 25th, 50th, and 75th percentiles.

**Figure 2:**
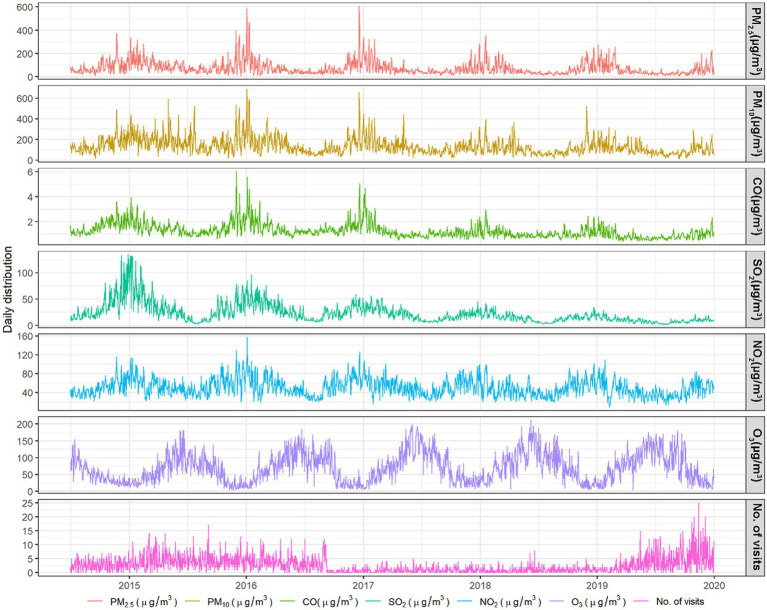
Time-series plot of daily outpatient visits for dry eye disease and corresponding daily air pollution concentrations in Zhengzhou, China, from July 1, 2014, to December 31, 2019.

Figure 2 illustrates the time-series patterns of air pollutants and dry eye visits throughout the study period. The air pollutants exhibited a distinct seasonal trend, with the lowest concentrations occurring during summer and the highest concentrations during winter.

**Figure 3:**
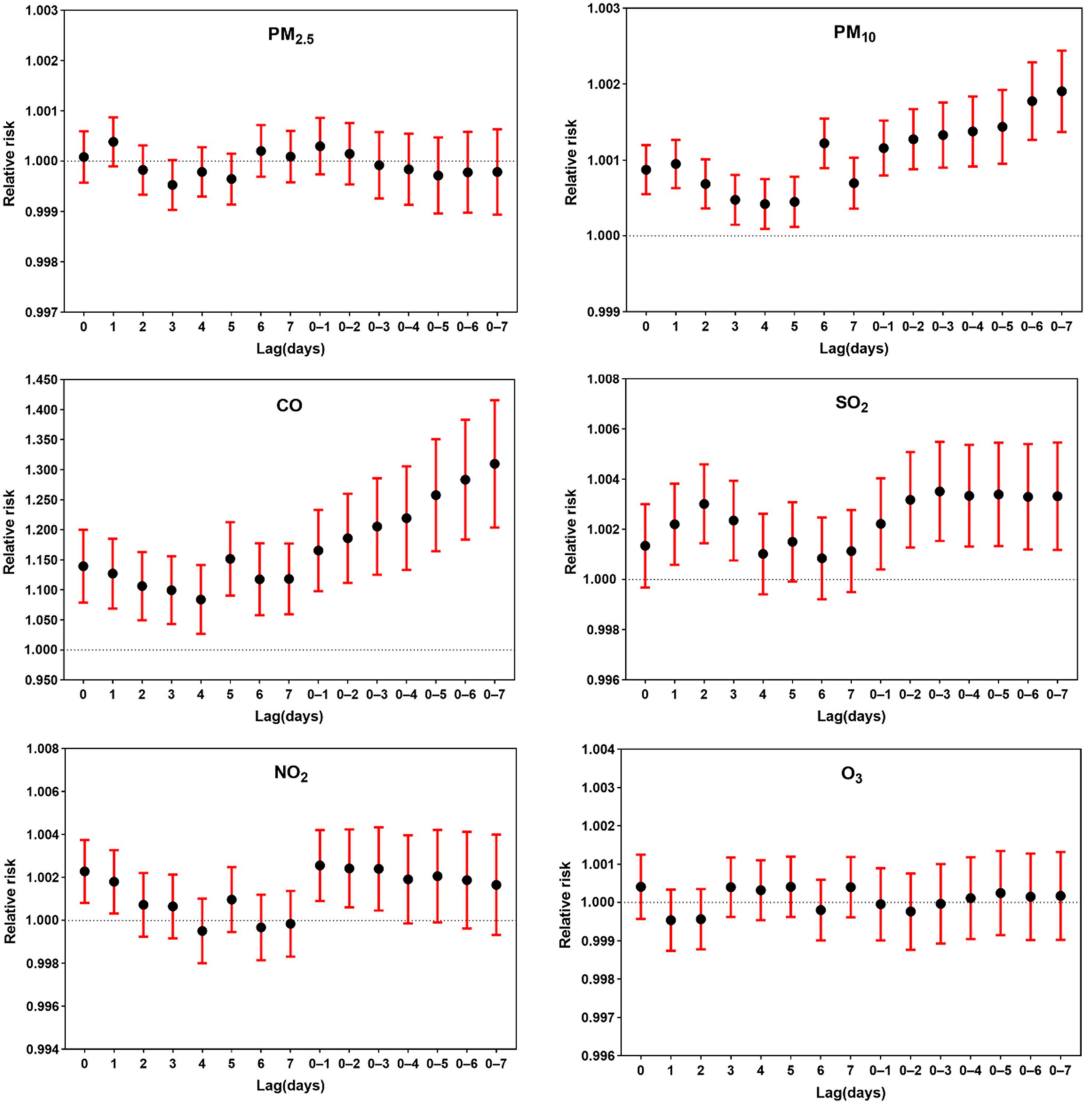
Relative risk of dry eye disease outpatient visits associated with a 10 μg/m^3^ increase in air pollutant levels across single-day lags (lag 0, 1, 2,…,7 days) and cumulative lags (lag 0–1, 0–2, 0–3,…, lag 0–7 days).

We analyzed the association between air pollutants and outpatient visits for DED using a single-pollutant model. We investigated the relationship between individual air pollutants and the number of outpatient visits for DED, with meteorological factors included as covariates. The specific meteorological factors considered were mean temperature, air pressure, relative humidity, and wind speed. Figure 3 demonstrates a significant association between a 10 μg/m^3^ increase in PM_10_, CO, SO_2_, and NO_2_ concentrations and outpatient visits for DED. Both PM_10_ and CO exhibited a significant association with an increase in DED visits for every 10 μg/m^3^ increase in concentrations, considering all lags from 0 to 7 days, in contrast to cumulative lags from 0–1 to 0–7 days. Significant associations were observed between SO_2_ and DED outpatient visits, considering lags of 1, 2, 3, 0–1, 0–2, 0–3, 0–4, 0–5, and 0–6 days, as well as between NO_2_ and DED outpatient visits, considering lags of 0, 1, 0–1, 0–2, and 0–3 days. The results of the cumulative lag (0–1 to 0–7 days) modeling suggest that continuous exposure to these air pollutants may lead to an increased number of DED visits. The analysis using the single-pollutant model did not provide evidence of an association between DED outpatient visits and PM_2.5_ or O_3_.

Considering the inevitable interactions between air pollutants, we employed multi-pollutant modeling for the analysis. Previous research has demonstrated a strong correlation between PM_2.5_ and PM_10_ (21). PM_10_ was included in the multi-pollutant model due to the lack of evidence for an association between PM_2.5_ and DED visits in the single-pollutant model analysis. The model incorporated meteorological variables as covariates, while pollutant levels within the optimal lag zone were selected as control variables. Even after adjusting for confounding factors, both PM_10_ and CO exhibited a strong association with DED outpatient visits, and the number of DED outpatient visits was also associated with NO_2_ levels. There was no significant association between the pollutants PM_2.5_, O_3_, and SO_2_ and DED outpatient visits. The lack of a significant association between SO_2_ and DED outpatient visits contrasts with the findings of the single-pollutant model (Table 2).

**Table 2:** Association between air pollutants and outpatient visits for dry eye disease: a multipollutant model.

Characteristics	PM_2.5_	PM_10_	CO	SO_2_	NO_2_	O_3_
Adjusted for PM_2.5_	_	_	1.2139(1.1268–1.3077)	1.0009(0.9992–1.0027)	1.0023(1.0005–1.0040)	1.0004(0.9996–1.0013)
Adjusted for PM_10_	_	_	1.0731(1.0135–1.1362)	1.0003(0.9986–1.0019)	1.0001(0.9993–1.0010)	1.0003(0.9994–1.0011)
Adjusted for CO	0.9997(0.9991–1.0002)	1.0007(1.0003–1.0010)	_	0.9998(0.9981–1.0016)	1.0012(0.9996–1.0027)	1.0007(0.9998–1.0015)
Adjusted for SO_2_	0.9997(0.9992–1.0003)	1.0008(1.0004–1.0011)	1.1118(1.0479–1.1797)	_	1.0014(0.9997–1.0030)	1.0004(0.9996–1.0013)
Adjusted for NO_2_	0.9997(0.9992–1.0003)	1.0008(1.0005–1.0012)	1.1340(1.0662–1.2060)	1.0011(0.9995–1.0028)	_	1.0006(0.9997–1.0014)
Adjusted for O_3_	1.0000(0.9995–1.0006)	1.0009(1.0005–1.0012)	1.1355(1.0759–1.1984)	1.0013(0.9996–1.0029)	1.0021(1.0006–1.0036)	_
Adjusted for the other 4 pollutants	0.9996(0.9991–1.0002)	1.0007(1.0004–1.0011)	1.0852(1.0172–1.1577)	0.9998(0.9980–1.0016)	1.0004(0.9987–1.0021)	1.0003(0.9995–1.0012)

Additionally, we examined the relationship between various air pollutants and DED outpatient visits across different population groups. Among males, elevated levels of PM_2.5_, PM_10_, CO, SO_2_, and NO_2_ were significantly linked to DED outpatient visits, while in females, only PM_10_ and CO showed significant associations with DED outpatient visits. Within the age group of 0–18 years, higher CO concentrations were significantly associated with DED outpatient visits. Within the age group of 19–59 years, elevated levels of PM2.5, PM10, and CO showed significant associations with DED outpatient visits. Within the age group of 60 and above, increasing concentrations of PM_10_, CO, and NO_2_ were significantly associated with outpatient visits for DED. Within the seasonal stratification, higher PM_10_ and CO concentrations during the warm season showed a significant association with DED outpatient visits. Moreover, increased levels of PM_10_, CO, SO_2_, and NO_2_ during the cool season were significantly associated with DED outpatient visits (Table 3).

**Table 3:** Correlation between air pollutants and outpatient visits for dry eye disease, and effect modification assessed through stratified analyses by patient characteristics.

Characteristics	PM_2.5_	PM_10_	CO	SO_2_	NO_2_	O_3_
Gender						
Male	1.0007(1.0001–1.0013)	1.0012(1.0008–1.0016)	1.1957(1.1168–1.2801)	1.0038(1.0017–1.0059)	1.0039(1.0020–1.0058)	1.0010(0.9999–1.0021)
Female	0.9997(0.9991–1.0003)	1.0007(1.0003–1.0010)	1.1067(1.0436–1.1737)	0.9999(0.9981–1.0018)	1.0013(0.9997–1.0029)	0.9999(0.9990–1.0008)
Age (years)						
0–18	0.9986(0.9972–1.0001)	0.9991(0.9983–1.0001)	1.2710(1.1134–1.4508)	0.9984(0.9940–1.0028)	0.9986(0.9947–1.0025)	1.0016(0.9994–1.0039)
19–59	1.0005(1.0000–1.0011)	1.0009(1.0005–1.0012)	1.0948(1.0351–1.1578)	1.0006(0.9989–1.0023)	1.0015(0.9999–1.0031)	1.0006(0.9997–1.0015)
60-	0.9997(0.9990–1.0004)	1.0009(1.0004–1.0013)	1.1402(1.0645–1.2213)	1.0020(0.9998–1.0042)	1.0032(1.0014–1.0050)	1.0003(0.9993–1.0012)
Season						
Warm season	1.0011(0.9997–1.0025)	1.0007(1.0002–1.0013)	1.1995(1.0499–1.3704)	0.9984(0.9935–1.0033)	0.9994(0.9967–1.0023)	1.0011(0.9999–1.0020)
Cold season	1.0002(0.9995–1.0008)	1.0010(1.0005–1.0015)	1.1311(1.0578–1.2095)	1.0032(1.0009–1.0056)	1.0021(1.0001–1.0041)	0.9985(0.9968–1.0002)

## 4 Discussion

This study found a significant association between air pollutants (PM_2.5_, PM_10_, CO, SO_2_, NO_2_) and outpatient visits for DED. A lag effect was observed in the potential association between PM_10_, CO, SO_2_, and NO_2_ and outpatient visits for DED. Among the gender stratified groups, females accounted for a larger proportion (63.76% of total study patients). Nevertheless, the association between air pollutants and the number of outpatient visits for DED was more pronounced in males compared to females. The association between air pollutants and the number of outpatient visits for DED varied across different age groups. The association between air pollutants and DED outpatient visits was more pronounced during the cold season compared to the warm season.

Previous studies have demonstrated the significant impact of air pollution on DED development, utilizing optical thickness measurements to assess aerosol concentrations in the atmosphere (22). Nevertheless, the association between air pollutants and DED exhibits variability across different studies. Discrepancies in study findings may arise due to variations in geographical regions, methodologies employed, and characteristics of the study populations. Our study revealed a significant association between a 10 μg/m^3^ increase in PM_10_ concentration and DED outpatient visits. Furthermore, PM_2.5_ exhibited a significant association with DED outpatient visits in males and the 19–59 years age group. It is worth noting that PM has the potential to induce ocular surface inflammation (23), which is considered a key component of DED in both humans (24) and animal models (25). Although most of the relevant studies, have found an association between PM and DED (3, 20, 26), there are individual studies that have shown no association between PM and DED (8, 18).

Similarly, not all studies have demonstrated an association between gaseous pollutants (SO_2_, NO_2_, CO, and O_3_) and DED (8, 26–28). However, our study identified significant associations between a 10 μg/m^3^ increase in CO, SO_2_, and NO_2_ concentrations and DED outpatient visits. This finding aligns with the results of a study conducted in Hangzhou, China (26), which did not identify an association between O_3_ and DED. The mechanisms underlying the potential impact of gaseous pollutants on ocular surface health are not fully comprehended. However, insights can be gleaned from extraneous data, which demonstrate that exposure to reactive gases outside the eye can induce cell death, oxidative stress, and inflammation (9, 29).

Our study differs from existing studies due to three potential reasons. Firstly, variations in climates and environments across different regions may have resulted in different effects of individual air pollutants on DED. Secondly, differences in study populations, along with variations in ocular surface physiology among populations, contribute to the complexity of clinical presentations. Thirdly, variations in study designs and methods can introduce bias into the results. In the present study, we employed GAM and time series modeling, with each patient serving as their own control. The model accounted for potential holiday effects, day of the week effects, and meteorological factors (e.g., mean temperature and mean humidity), ensuring more accurate results. In conclusion, our study provides additional evidence supporting the role of air pollution in the increasing prevalence of DED.

In the lag analysis of the single-pollutant model, the peak effects on DED outpatient visits were observed at specific lags: PM_10_ at lag 6, CO at lag 5, SO_2_ at lag 2, and NO_2_ at lag 1 (Figure 3). The lag effect on DED patients may be part of the underlying mechanism for the development of dry eye. The ocular surface has a certain level of tolerance to environmental changes, and only when this tolerance threshold is exceeded, the stability of the tear film on the ocular surface decreases, resulting in various ocular discomforts. Cumulative lag effect analysis revealed a more pronounced cumulative lag effect of PM_10_ and CO on the number of DED outpatient visits. The effect gradually increased from lag 0–1 to lag 0–7, indicating that continuous exposure to high concentrations of PM_10_ and CO may contribute to an increased number of DED outpatient visits.

Studies have consistently demonstrated that women are more susceptible to DED compared to men, and sex hormones may act as predisposing factors for the condition. Androgens enhance the function of the meibomian glands by promoting oil secretion onto the ocular surface, thus preventing tear evaporation. However, the effects of estrogen and progesterone on meibomian gland function may counteract the influence of androgens (30). While the precise effects of estrogen and progesterone on the ocular surface are not yet fully understood, estrogen has been implicated in contributing to inflammation of the ocular surface. Therefore, this study further supports the notion that women are a susceptible population for DED.

Air pollution may have indirect effects on the eyes. For example, fine particulate matter from air pollution can enter the lungs and cause an inflammatory response, which in turn affects circulation and the immune system throughout the body. These changes may have a negative impact on the health of the eye surface. A study in Latin America looked at the effects of air pollutants in multiple cities on cardiovascular and respiratory mortality in adults. The study showed that the increase in mortality risk was greater in men across gender groups, and that the effects of air pollutants on the cardiovascular and respiratory systems may be greater in men (31). Relatively few studies have addressed the relationship between air pollution, ocular surface disease and gender differences. Our study showed that the association between air pollutants and outpatient visits for DED was stronger in men than in women. This may be related to male lifestyle and behavioral habits, for example, men are more likely to engage in outdoor work and activities, thus increasing eye exposure to pollutants. This reason is only speculative, and the interpretation of this result in this study lacks some supporting evidence, and further studies are needed to explore in depth the gender-differentiated effects of air pollution on ocular surface diseases and their mechanisms.

The lifestyle habits of different age groups vary considerably, which may explain the difference in outpatient visits for DED and associated air pollutants for different age groups. Children’s activities are mainly indoors (home or classroom), and air pollutant data from urban monitoring sites were used in this study, so the results showed fewer air pollutants associated with them. DED patients in the 0–18 years age group are more likely to be associated with the overuse of electronic devices in modern society (32) or the habit of wearing contact lenses in the younger age group (33). Older individuals may be more sensitive to air pollution, thus increasing the risk of dry eye (34). But the intrinsic reasons for the difference in outpatient visits for DED and associated air pollutants for different age groups are not clear, and we need more studies to prove it. Of course, ecological studies can only provide a hypothesis for the association, and it cannot be ruled out that different studies may yield different results, so the results should be interpreted with caution.

In the seasonal stratified analysis, air pollutants (PM_10_, CO, SO_2_, and NO_2_) showed a significant association with DED outpatient visits during the cool season. During the warm season, only PM_10_ and CO exhibited a significant association with DED. This may be attributed to the increased sensitivity of the ocular surface to air pollutants in colder temperatures. Higher outdoor humidity demonstrated a protective association with DED symptoms (8), whereas wind speed and sunshine hours were identified as risk factors for DED diagnosis (35). Zhengzhou city experiences a warm temperate continental climate characterized by dry, cold winters with limited precipitation, as well as low rainfall and wind in spring. Hence, the unique climate conditions contribute to a stronger association between air pollutants and the number of DED outpatient visits during the cold season.

This study had several limitations. Firstly, it was designed as an ecological study, which only allows for drawing conclusions regarding possible associations, limiting the ability to make causal inferences. Secondly, the measured air pollutant concentrations at air quality monitoring stations may not accurately represent individual exposure levels. Future cohort studies assessing air pollution exposure are necessary to further validate the impact of air pollution on eye health. Lastly, the clinical data used in this study were obtained solely from a single large hospital, which is representative but has inherent limitations. The influencing factors of DED are complex. In the future, our goal is to conduct more in-depth analyses using different research designs such as case–control studies and cohort studies. These approaches will allow us to accurately assess individual exposure levels and thoroughly consider various factors, including lifestyle, age, gender, and medical history.

## 5 Conclusion

This study investigated the relationship between outpatient visits for DED and air pollution levels in Zhengzhou, China. The results demonstrated significant associations between DED outpatient visits and PM_2.5_, PM_10_, CO, SO_2_, and NO_2_. These associations exhibited gender and seasonal variations, with a particularly strong association observed during the cold season, with males being the most susceptible. While air pollution may serve as a potential risk factor for DED, our findings establish an association without establishing causation. Considering the rapid economic and industrial development in the region, implementing robust environmental protection strategies to mitigate air pollution is crucial for safeguarding eye health.

## Data availability statement

The raw data supporting the conclusions of this article will be made available by the authors, without undue reservation.

## Ethics statement

Ethical approval was not required for the study involving humans in accordance with the local legislation and institutional requirements. Written informed consent to participate in this study was not required from the participants or the participants’ legal guardians/next of kin in accordance with the national legislation and the institutional requirements.

## Author contributions

MX: Investigation, Software¸ Writing – original draft. YY: Investigation, Methodology, Software, Writing – original draft. JS: Data curation, Investigation, Methodology, Writing – review & editing. RH: Investigation, Methodology, Writing – original draft. YH: Formal analysis, Investigation, Writing – original draft. MZ: Methodology, Software, Writing – review & editing. XY: Data curation, Resources, Supervision, Writing – review & editing.

## Footnotes

^1^ http://data.cma.cn/
